# Modeling college EFL teachers’ intentions to conduct academic research: Integrating theory of planned behavior with self-determination theory

**DOI:** 10.1371/journal.pone.0307704

**Published:** 2024-08-27

**Authors:** Xiaobin Ren

**Affiliations:** School of Foreign Languages, Guangxi University, Nanning, China; Bahir Dar University, ETHIOPIA

## Abstract

This study constructed a robust theoretical model aimed at elucidating the determinants that shape college EFL teachers’ research intentions by integrating the tenets of Self-Determination Theory (SDT) with Theory of Planned Behavior (TPB). This model was empirically validated using data from 271 EFL teachers from eight colleges in China, selected through stratified sampling and collected via paper questionnaires, then analyzed using structural equation modeling. The findings underscore the instrumental roles of both autonomous and controlled motivations in driving research-related behaviors, thereby reinforcing the foundational concepts of SDT. Additionally, this study provides intricate insights into the mechanisms wherein motivation steers immediate determinants of research intention, encompassing attitudes, subjective norms, and perceived behavioral control. This melding of SDT and TPB offers an all-encompassing perspective on the multifaceted nexus between motivation and research intentions. Consequently, this refined model not only marks a pivotal stride in advancing teacher education theory but also establishes a guiding framework for forthcoming research and interventions, accentuating the imperative of fostering research intentions among college EFL educators.

## 1. Introduction

In recent years, universities globally have intensified their focus on research activities with aspirations of elevating their academic standings and overall competitiveness [1, 2]. This shift has placed college EFL teachers, among other academic faculty, under magnified pressures to produce research outputs, which have come to be recognized as pivotal metrics for performance evaluations [3]. Beyond its evaluative implications, research engagement offers EFL teachers the bridge that connects pedagogical theories to classroom practices, fostering a richer, evidence-based understanding of their teaching methods [4, 5]. Yet, the escalating demands for scholarly outputs can be daunting due to factors such as time constraints [6], lack of resources [7, 8], and the challenge of balancing teaching and research roles [9].

A review of literature reveals an abundance of studies probing into EFL teachers’ motivations and intentions to teach [e.g., 10, 11], engage in community service [e.g., 12–14], use digital teaching tools [e.g., 15, 16], and involve in professional development activities [e.g., 17, 18], yet there remains a conspicuous scarcity of research focused on their motivations and intentions behind academic research [6]. In reality, many EFL teachers exhibit limited research intentions and motivations [3, 4], compounded by insufficient research capacity [19] and fragile research environment [20], which consequently results in constrained research output [1]. This oversight of research motivation and intention is especially salient considering the increasing demands on EFL educators to contribute research to their fields. Furthermore, understanding what propels these educators toward research is pivotal, not only for their individual professional development but also for the broader elevation of English education standards.

In light of this, our study aims to construct a research model that delineates the intricate factors influencing college EFL teachers’ intentions toward academic research. Drawing upon the robust principles of the Self-Determination Theory (SDT) and the Theory of Planned Behavior (TPB), and by employing questionnaires and structural equation modeling, we aspire to chart out a comprehensive and applicable framework that can serve as a beacon for understanding and nurturing research inclinations amongst college EFL educators. By identifying key motivational and behavioral determinants, our findings can provide academic institutions and policymakers with actionable insights to cultivate research competencies and foster research-oriented environments. Specifically, these tools can be utilized to design targeted professional development programs, allocate resources effectively, and create supportive policies that alleviate barriers to research engagement, ultimately enhancing the research output and academic standing of institutions.

## 2. Literature review and hypotheses formulation

### 2.1 EFL teachers’ academic research behaviors

A myriad of investigations has dissected the motivations of EFL teachers across different educational hierarchies [11, 21, 22]. Nonetheless, a disproportionate focus remains on their motivation to teach English rather than engage in academic research [10, 11, 21]. This imbalance in research focus highlights a significant gap in understanding the factors that drive EFL teachers to undertake academic research. Teaching and research are critical tasks for most college English teachers globally, yet the motivations driving these activities are distinct and influenced by different sets of factors [23]. While teaching motivation is often driven by immediate classroom dynamics, student engagement, and pedagogical objectives [24], research motivation is often shaped by professional development, instrumental incentives or institutional expectations [25]. Understanding this distinction is crucial, as strategies that enhance teaching effectiveness do not necessarily foster research productivity.

Besides, although there exists some research zoning in on reasons that prompt or hinder research activities [e.g., 4, 6], a comprehensive understanding of the multifaceted interplay influencing EFL teachers’ research intentions remains elusive. As such, a holistic framework capturing the interconnected internal dynamics and the pivotal role of motivation in steering college EFL teachers’ research inclinations is conspicuously needed. This study aims to develop such a framework by leveraging two prominent theoretical models, the Theory of Planned Behavior and Self-Determination Theory, to provide valuable insights for fostering research inclinations among college EFL educators.

### 2.2 Theory of planned behavior

The theory of planned behavior (TPB) evolved from the theory of reasoned action (TRA), which posited that attitudes and subjective norms dictated behavioral intentions [26]. TRA’s limited scope, assuming behaviors were mainly volitional [27], prompted Ajzen (1985) to introduce “perceived behavior control (PBC)” from Bandura [28]’s self-efficacy theory, broadening its applicability. Thus, TPB encompasses four constructs: behavioral intention, attitudes, subjective norms, and PBC.

#### 2.2.1 Behavioral intention

The construct of behavioral intention is a fundamental concept in TPB. It refers to the degree to which an individual is willing to exert effort to engage in a particular behavior [27, 29]. In the context of this study, behavioral intention is defined as college EFL teachers’ willingness to conduct research. Understanding the factors that influence college EFL teachers’ research intentions can therefore provide valuable insights for promoting research engagement among this group.

#### 2.2.2 Attitude towards research

Attitude, as defined by Ajzen [30], refers to the degree to which an individual evaluates a behavior as favorable or unfavorable. In the context of this study, attitude towards research refers to the extent to which college EFL teachers evaluate conducting research as a desirable and worthwhile activity. Before forming an intention to conduct research, college EFL teachers tend to have positive or negative attitudes towards this behavior. A positive attitude towards conducting research may be formed when it is believed to have favorable and desirable outcomes. Therefore, the more advantageous and favorable conducting research is perceived, the more intense the intention to invest in it [31]. Previous studies have consistently shown that academic staff’s attitudes towards research have a significant influence on their research-related intentions [e.g., 32–35]. Based on these concerns, the following hypothesis is proposed:

H1a: College EFL teachers’ attitudes towards research have a significant impact on their intention to conduct research.

#### 2.2.3 Subjective norms

Subjective norms refer to the social pressure that individuals perceive for performing a behavior [27, 29]. These pressures can come from close communities or influential people, such as parents, spouses, friends, colleagues, and students. In the context of this study, subjective norms refer to the pressure that college EFL teachers sense to conduct academic research. Previous studies have found the influence of subjective norms on people’s academic intentions. For instance, Khuram, Wang [35] found that subjective norms had a significant influence on international doctoral students’ knowledge-seeking intentions in Chinese universities. Borg and Liu [3] found that personal and contextual factors, such as policy requirements and colleague support, were related to EFL teachers’ motivation and demotivation to conduct research. Therefore, it is hypothesized that:

H1b: College EFL teachers’ subjective norms towards research have a significant impact on their intention to conduct research.

#### 2.2.4 Perceived behavior control

Perceived behavior control (PBC) means the extent of ease or difficulty sensed by an individual in terms of conducting a particular behavior [36]. Generally, individuals are more likely to engage in an activity if they perceive it to be easy, and less likely to invest in it if they perceive it to be difficult [37]. In this study, PBC refers to the degree of ease or difficulty college EFL teachers sensed for conducting academic research. PBC has been identified as a significant predictor of people’s academic-related intentions in previous studies. For example, Manstead and Van Eekelen [38] found students’ academic achievement intentions could be predicted by their PBC. Similarly, Ponnet, Wouters [39] found that PBC was a predictor of college students’ intentions to use stimulants for academic performance enhancement. Based on this concern, it is hypothesized that:

H1c: College EFL teachers’ PBC towards research has a significant impact on their intention to conduct research.

The theory of planned behavior (TPB) is widely used to explain various behaviors, yet its ability to predict EFL teachers’ research intentions remains underexplored. Some critiques, like Hagger and Chatzisarantis [40], argue that TPB doesn’t distinguish between sought-after results and obligatory ones. Others, such as Luqman, Masood [41], believe TPB does not fully capture intentional behavior dimensions, emphasizing the need for recognizing reasons like autonomous or controlled motives. These critiques suggest integrating self-determination theory (SDT) to bolster TPB’s predictive capacity.

### 2.3 Self-determination theory

**Fig 1:**
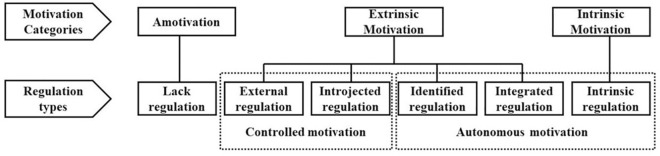
Motivation categories according to SDT (this figure was drawn by drawing upon the figures in Kunz [45]’s and Hu, Tang [46]’s research).

SDT [42] is a comprehensive theory that explains human motivation and development, and it has been extensively studied and applied across various domains, including entrepreneurship, health, and sports [43]. According to Deci and Ryan [42], self-determination refers to individuals’ independent decision-making based on a thorough understanding of their needs and environmental information. SDT classifies motivation based on quality rather than quantity, grouping individuals’ behaviors into three categories: amotivation, extrinsic motivation, and intrinsic motivation. Amotivation arises from a sense of incompetence and helplessness when faced with an activity (e.g., EFL teachers feeling inadequate and disinterested in research). Extrinsic motivation comprises four subtypes by Ryan and Deci [44]: external regulation (driven by external rewards or punishments, such as bonuses or penalties), introjected regulation (guided by internal rewards or punishments like guilt or self-esteem protection), identified regulation (based on understanding an activity’s value, such as enhancing teaching skills and career prospects), and integrated regulation (where activity values are internalized, such as internalizing the value of research activity into personal and professional identity). Intrinsic motivation is the desire to engage in a behavior for pleasure and satisfaction (e.g., interest and enjoyment in exploring new linguistic theories). Controlled motivation (external and introjected regulations) arises from external pressures or self-esteem protection, while autonomous motivation (identified, integrated regulations, and intrinsic motivation) arises from internal values or satisfaction. This motivation spectrum is illustrated in Fig 1.

In this study, controlled motivation refers to the extent to which college EFL teachers engage in academic research driven by external pressures, rewards, or internal compulsions, such as guilt or the need to maintain self-esteem. This type of motivation is influenced by factors outside the individual or by internal pressures to comply with certain expectations. On the other hand, autonomous motivation is defined as the degree to which EFL teachers are driven to conduct research based on their internal values, genuine interest, and personal satisfaction derived from the activity itself. This motivation arises from a sense of volition and self-endorsement of the research activities, reflecting a deeper personal commitment and intrinsic enjoyment in the research process.

Although SDT categorizes individuals’ motivation, thus avoiding the limitation of TPB, this theory does not clearly elaborate how proximal factors (e.g., people’s beliefs, perceptions of control) impact their behavioral intentions [47]. Therefore, integrating the model of SDT and TPB might overcome the shortcomings of the two theoretical frameworks and provide a more comprehensive understanding of individuals’ motivational and cognitive processes. Currently, several studies have been conducted integrating SDT and TPB as a theoretical framework and found autonomous and controlled motivation could function as distal predictors of intention in the integrated model, while attitude, subjective norms, and PBC could be considered as proximal factors for intention [e.g., 41, 47, 48]. Based on these concerns, it is hypothesized that:

H2: College EFL teachers’ autonomous motivation to conduct research has a significant impact on their attitudes (H2a), subjective norms (H2b), and PBC (H2c) towards research.
H3: College EFL teachers’ controlled motivation to conduct research has a significant impact on their attitudes (H3a), subjective norms (H3b), and PBC (H3c) towards research.

**Fig 2:**
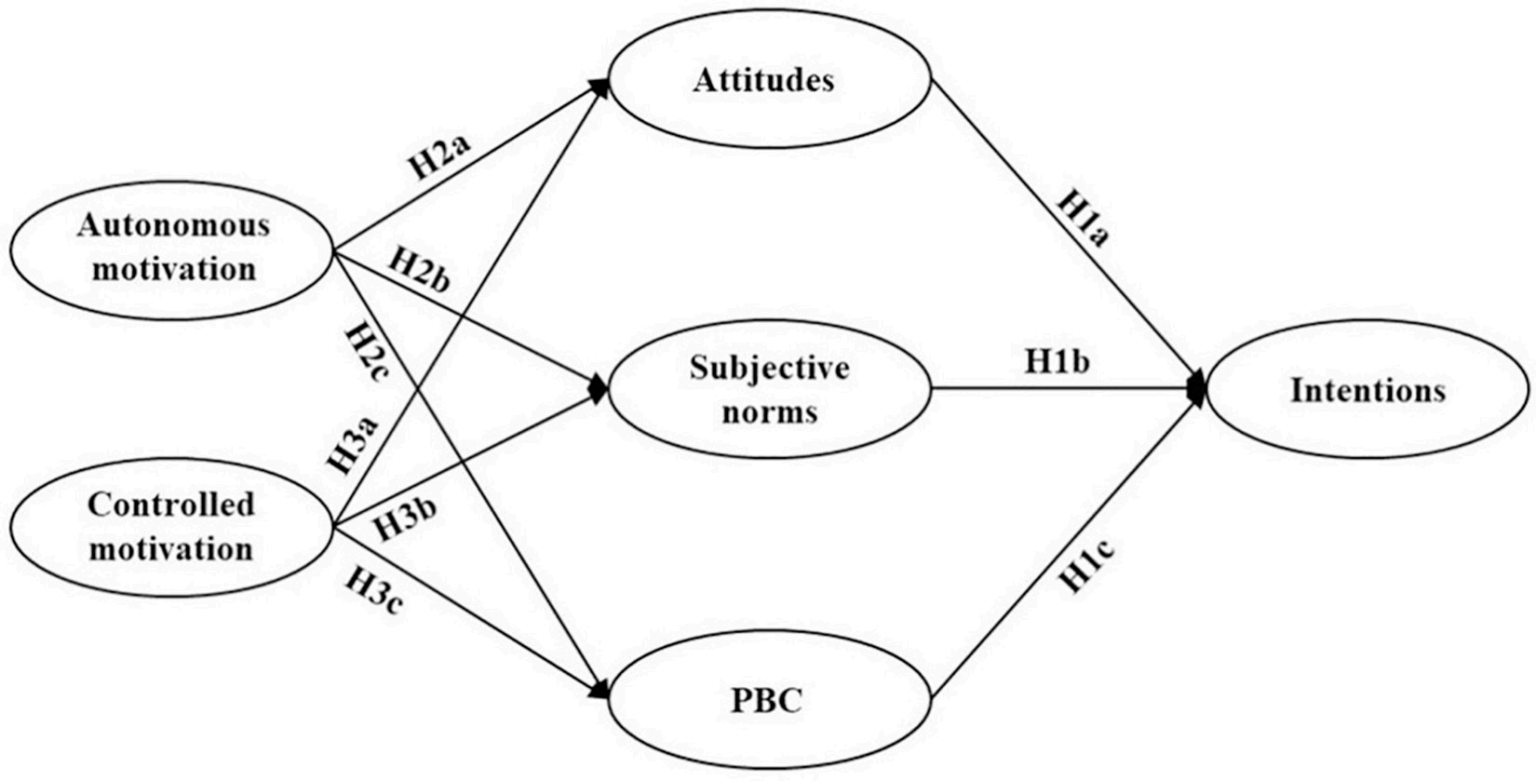
Research model and hypotheses.

The research model in Fig 2 depicts these hypothesized relationships.

## 3. Methods of the study

This study employed a quantitative survey research design to elaborate the determinants that shape college EFL teachers research intentions.

### 3.1 Research context

In this study, academic research is characterized by systematic inquiry and investigation into specific areas or topics within the broader field of English studies. Such areas might span English linguistics, literature, and applied linguistics, among others. This involves activities such as conducting experiments, exploring new methodologies, writing and publishing scholarly articles, applying for research grants, presenting at conferences, engaging in collaborative projects, and other behaviors contributing to academic communities. These academic endeavors aim to contribute to the wider academic discourse, offer fresh insights, and push forward the current understanding in the respective fields.

It’s important to note that while pedagogical reflections and the honing of teaching skills are valuable in shaping teaching practices and are indeed a form of research [49, 50], they do not fall under the scope of “academic research” as defined in this study. Our emphasis lies primarily on the scholarly activities that contribute to the academic community at large, especially those leading to formal recognition, such as publications in esteemed journals or acquisition of research grants. By including a broader range of scholarly activities, we aim to capture the full spectrum of academic research efforts undertaken by college EFL educators.

### 3.2 Participants

In Chinese colleges, EFL teachers can be categorized into two distinct groups. The first group comprises teachers responsible for instructing non-English major college students, while the second group caters to English major students [51]. Despite the variance in their target student audience, both groups are required to showcase tangible research outcomes when it comes to professional advancement and career progression. Such outcomes span a range of scholarly endeavors, including but not limited to, the publication of research papers, authoring academic books, and successfully executing research projects [6]. Thus, irrespective of the differences in their teaching cohort, engaging in academic research remains a consistent and imperative undertaking for all EFL teachers in Chinese higher education institutions. This study did not differentiate between EFL teachers instructing English majors and non-English majors; both groups were included in the research sample. Finally, a total of 298 college English teachers, including those teaching English majors and non-English majors, participated in this study, providing a comprehensive dataset for analysis.

### 3.3 Instruments

Questionnaires were applied in this study to collect data from college EFL teachers. To ensure the reliability and validity of the variables, the items in the questionnaire were drawn upon from those frequently adopted ones in previous studies and were adjusted according to related studies on college EFL teachers’ intention to conduct research. In addition, to improve the content validity of the items, the questionnaire was also reviewed and revised according to two PhD-holding EFL teacher experts from different universities, both specializing in questionnaire design and application in academic studies. For example, the item “Among various options, I’d rather be a teacher-researcher” was considered by one expert but cannot be used as an item for “Attitude” variable and therefore was deleted. Then, a pilot study, involving 52 participants, was conduct before the questionnaires were handed out to the large group of EFL teachers, and 2 items were deleted because of low CITC values (<0.5), as suggested by Ma [52]. Excluding the above 2 items, all variables had Cronbach’s α values above .70 (see Table 1), indicating the questionnaire had reasonable reliability and could be used in further study.

**Table 1:** Reliability test results in pilot study.

Variables	ATM	CTM	ATT	SN	PBC	INT
Alpha	.784	.890	.911	.794	.806	.924

The questionnaire used 7-point Likert scale with 1 representing “strongly disagree” while 7 representing “strongly agree”. The final questionnaire included 21 items measuring the 6 constructs: autonomous motivation (4 items) and controlled motivation (4 items), and the four TPB constructs: attitudes (3 items), subjective norms (3 items), PBC (3 items), and intentions (4 items). Table 2 demonstrated the details of measuring items.

**Table 2:** Measurement instrument.

Latent constructs	Observable constructs	Adapted construct items	Sources
Autonomous motivation	ATM 1	I enjoy the work of researching.	[45, 53, 54]
	ATM 2	I conduct research for the promotion of my professional title.	
	ATM 3	Conducting research fits my personal values.	
	ATM 4	I conduct research for the pleasure of exploring.	
Controlled motivation	CTM 1	I conduct research because otherwise I will be punished by our university.	[45, 53, 54]
	CTM 2	I conduct research because otherwise I will be regarded as an unqualified college teacher.	
	CTM 3	I conduct research to get the incentives from university.	
	CTM 4	I conduct research because otherwise I would feel guilty for not doing them.	
Attitudes	ATT 1	A career as a teacher-researcher is attractive for me.	[37, 41, 55]
	ATT 2	Being a teacher-researcher could give great satisfactions to me.	
	ATT 3	Being a teacher-researcher implies more advantages than disadvantages.	
Subjective norms	SN 1	Most of my colleagues think conducting research is important for our career.	[37, 55]
	SN 2	University gives us pressure and requirements to conduct research.	
	SN 3	The leaders in my department think I should conduct research.	
PBC	PBC 1	I have the resources necessary to conduct research.	[37, 41, 55]
	PBC 2	I know the necessary practical procedures to conduct research.	
	PBC 3	If I tried to conduct research, I would have a high probability of being productive.	
Intentions	INT 1	I would conduct academic research in the future.	[37, 55]
	INT 2	I’m ready to make anything to be a teacher-researcher.	
	INT 3	I have very seriously thought in becoming a teacher-researcher.	
	INT 4	I’ve got the firm intention to conduct academic research.	

### 3.4 Sampling techniques and procedures

Stratified sampling technique [56] was chosen in this study to ensure the sample accurately represents the diversity of the target population. The strata were defined based on the type of institution (public vs. private). According to the Ministry of Education of People’s Republic of China [57], private colleges account for 25.36% of the total higher-education institutions in China. Based on stratified sampling technique and proportion of public-private higher education institutions, this study selected EFL teachers from 8 colleges, including 6 public and 2 private institutions, all located in a central province in China. The distribution in this study, with 25% private and 75% public universities, aligned with this national percentage, ensuring proportional representation.

Invitation messages were sent to the heads of schools to obtain their permission to distribute the questionnaire to their faculty during the first semester of the 2023–2024 academic year (approximately from September to December 2023). Upon receiving approval from the heads, the researcher distributed the questionnaires during their regular meetings. A total of 351 paper questionnaires were distributed to EFL teachers in the selected colleges over a period of three months. All participating teachers were assured that their responses would remain confidential. The original response rate was approximately 76%, which is acceptable but still leaves room for non-response bias. To mitigate this, follow-up reminders were sent to increase participation. Finally, 298 questionnaires were returned (response rate 84.9%), out of which 271 were deemed valid for analysis. The demographic information of the respondents is presented in Table 3.

**Table 3:** Demographics of the respondents.

Demographics		F	%
Gender	Male	46	16.97
	Female	225	83.03
Age	21–30	53	19.56
	31–40	101	37.27
	41–50	79	29.15
	51–60	38	14.02
Working experience	0–10	115	42.44
	11–20	97	35.79
	>20	59	21.77
University type	Public	187	69.00
	Private	84	31.00
Degree	PhD	48	17.71
	Master	214	78.97
	Bachelor	9	3.32

### 3.5 Data analysis

The questionnaire data were input into SPSS 25 and Amos 22, and analyzed quantitatively using descriptive statistics and inferential statistics. Among descriptive statistics, mean and standard deviation were used whereas correlation, goodness-of-fit indices, path coefficients, and reliability and validity indices were used in inferential statistics.

### 3.6 Statement

This study was approved by Medical Ethics Committee of Guangxi University (No.: GXU-2023-024). Written informed consent was obtained from all participants prior to data collection, ensuring that they were fully aware of the study’s purpose, procedures, and any potential risks or benefits. Ethical considerations, including confidentiality and the right to withdraw from the study at any time, were strictly adhered to.

## 4. Results

To ensure the validity of the structural equation modeling (SEM) analysis, we assessed the normality and multicollinearity of the data. Visual inspections of histograms indicated that each observed variable approximated a normal distribution. Additionally, the correlation coefficients among the latent variables ranged from 0.3 to 0.7, suggesting moderate correlations and indicating that multicollinearity is not severe [58]. These findings confirm that the data meet the necessary assumptions for SEM analysis. To test the internal consistency of every variable, reliability test was conducted, and the results could be found in Table 4. As suggested by Cheung, Cooper-Thomas [59], this study reports both Cronbach’s Alpha and McDonald’s Omega for each variable to ensure reasonable reliability. All the Cronbach’s Alphas and McDonald’s Omegas for the six variables were above .70, indicating that the questionnaire had reasonably high reliability.

**Table 4:** Reliability test results.

Variables	ATM	CTM	ATT	SN	PBC	INT
Alpha	.814	.808	.882	.759	.729	.890
Omega	.815	.808	.883	.767	.730	.893

Note: ATM = Autonomous motivation; CTM = Controlled motivation; ATT = Attitudes; SN = Subjective norms; PBC = Perceived behavior control; INT = Intention to conduct research.

Convergent validity test results were demonstrated in Table 5. All the CRs of the six variables were above 0.7 and almost all AVEs were above 0.5 (AVE for PBC was very near 0.5), indicating that the convergent validity of the six variables were acceptable.

**Table 5:** Convergent validity test results.

Variables	Items	Unstd.	S.E.	*t*-value	*p*	Std.	SMC	CR	AVE
ATM	ATM1	1.000				.744	.554	.817	.529
	ATM2	1.041	.092	11.266	***	.803	.645		
	ATM3	.911	.095	9.631	***	.645	.416		
	ATM4	.916	.090	10.187	***	.708	.501		
CTM	CTM1	1.000				.699	.489	.811	.518
	CTM2	.980	.093	10.537	***	.780	.608		
	CTM3	.916	.094	9.707	***	.725	.526		
	CTM4	.868	.095	9.179	***	.671	.450		
ATT	ATT1	1.000				.828	.686	.887	.724
	ATT2	1.116	.067	16.586	***	.941	.885		
	ATT3	.990	.068	14.572	***	.775	.601		
SN	SN1	1.000				.683	.466	.770	.533
	SN2	1.266	.154	8.222	***	.881	.776		
	SN3	.891	.105	8.453	***	.598	.358		
PBC	PBC1	1.000				.676	.457	.731	.475
	PBC2	1.122	.148	7.568	***	.729	.531		
	PBC3	1.084	.142	7.629	***	.662	.438		
INT	INT1	1.000				.637	.406	.897	.689
	INT2	1.279	.110	11.594	***	.850	.723		
	INT3	1.357	.110	12.290	***	.940	.884		
	INT4	1.302	.111	11.704	***	.861	.741		

Note: ATM = Autonomous motivation; CTM = Controlled motivation; ATT = Attitudes; SN = Subjective norms; PBC = Perceived behavior control; INT = Intention to conduct research.

***indicates *p* < .001.

Discriminate validity test demonstrated that the square root of every variable’s AVE was higher than the Pearson correlations between the corresponding construct and others (see Table 6), indicating the hypothesized models had acceptable discriminate validity.

**Table 6:** Discriminate validity test results.

	AVE	INT	PBC	SN	ATT	CTM	ATM
INT	.689	**.830**					
PBC	.475	.515	**.689**				
SN	.533	.465	.406	**.730**			
ATT	.724	.465	.396	.417	**.851**		
CTM	.518	.508	.598	.551	.418	**.720**	
ATM	.529	.375	.573	.517	.424	.594	**.727**

Note: ATM = Autonomous motivation; CTM = Controlled motivation; ATT = Attitudes; SN = Subjective norms; PBC = Perceived behavior control; INT = Intention to conduct research.

Square roots of AVEs are in bold on diagonal, while off diagonal are Pearson correlations of variables.

**Fig 3:**
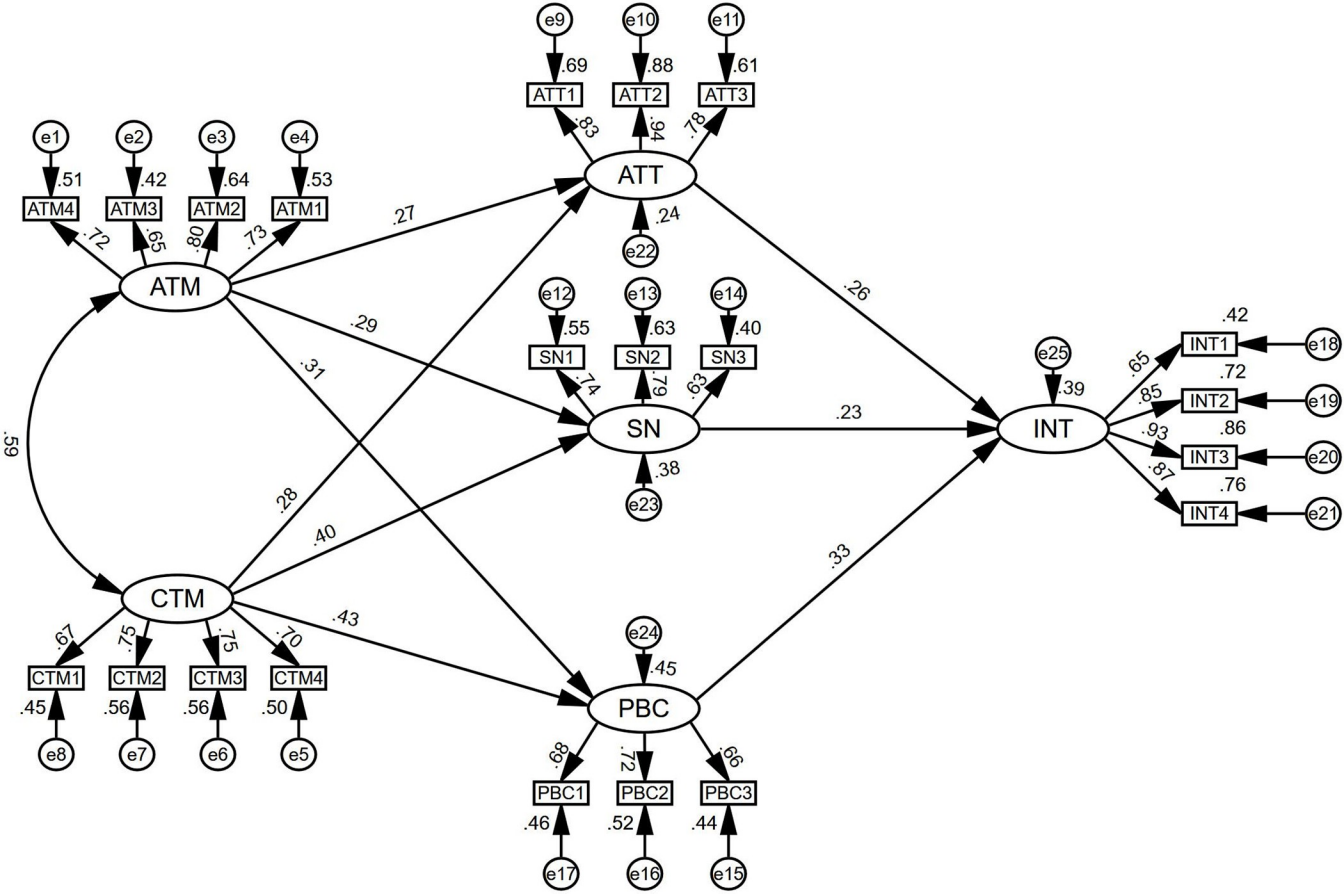
Research model with factor loadings.

Table 7 displayed the model fit results for the hypothesized model. The values of GFI, AGFI, CFI and TLI were all above 0.9. In addition, χ^2^/df, RMSEA, and SRMR also indicated that the hypothesized model was acceptable. The research model with factor loadings was demonstrated in Fig 3.

**Table 7:** Model fit results.

Indexes	Values	Suggested values
*χ2*	242.345	---
*χ2*/*df*	1.339	<3.0
GFI	.923	>.90
AGFI	.902	>.90
CFI	.977	>.90
TLI	.973	>.90
RMSEA	.035	< .08
SRMR	.049	< .05

Table 8 presents the results of the hypotheses testing for the 9 paths in the hypothesized model. The table includes both the standardized and unstandardized path coefficients, along with their respective standard errors (S.E.), critical ratios (C.R.), and significance levels.

**Table 8:** Results of hypotheses testing.

Hypotheses			Unstd.	S.E.	C.R.	Std.	Results
ATT	<---	ATM	0.344**	0.115	3	0.269	Accepted
SN	<---	ATM	0.33**	0.107	3.083	0.287	Accepted
PBC	<---	ATM	0.392**	0.121	3.249	0.314	Accepted
ATT	<---	CTM	0.325**	0.103	3.153	0.281	Accepted
SN	<---	CTM	0.413***	0.103	4.007	0.398	Accepted
PBC	<---	CTM	0.487***	0.115	4.232	0.432	Accepted
INT	<---	ATT	0.211***	0.055	3.826	0.256	Accepted
INT	<---	SN	0.216**	0.069	3.126	0.235	Accepted
INT	<---	PBC	0.282***	0.07	4.042	0.332	Accepted

Note: ATM = Autonomous motivation; CTM = Controlled motivation; ATT = Attitudes; SN = Subjective norms; PBC = Perceived behavior control; INT = Intention to conduct research.

***indicates *p* < .001

** indicates *p* < .01.

The following section is a detailed explanation of each path, demonstrating the significance and impact of various factors related with college EFL teachers’ research intentions.

H1a (INT ← ATT): The path from attitudes (ATT) to intentions (INT) was significant (Unstd. = 0.211, Std. = 0.256, *p* < .001), demonstrating that positive attitudes towards research significantly predict the intention to engage in research.

H1b (INT ← SN): The path from subjective norms (SN) to intentions (INT) was significant (Unstd. = 0.216, Std. = 0.235, *p* < .01), indicating that perceived social pressure significantly influences research intentions.

H1c (INT ← PBC): The path from perceived behavioral control (PBC) to intentions (INT) was significant (Unstd. = 0.282, Std. = 0.332, *p* < .001), showing that greater perceived control over research activities significantly predicts the intention to conduct research.

H2a (ATT ← ATM): The path from autonomous motivation (ATM) to attitudes (ATT) was significant (Unstd. = 0.344, Std. = 0.269, *p* < .01), indicating that higher levels of autonomous motivation positively influence teachers’ attitudes towards research.

H2b (SN ← ATM): The path from autonomous motivation (ATM) to subjective norms (SN) was significant (Unstd. = 0.33, Std. = 0.287, *p* < .01), suggesting that autonomous motivation positively affects the perceived social pressure to engage in research.

H2c (PBC ← ATM): The path from autonomous motivation (ATM) to perceived behavioral control (PBC) was significant (Unstd. = 0.392, Std. = 0.314, *p* < .01), showing that autonomous motivation enhances teachers’ confidence in their ability to conduct research.

H3a (ATT ← CTM): The path from controlled motivation (CTM) to attitudes (ATT) was significant (Unstd. = 0.325, Std. = 0.281, *p* < .01), indicating that controlled motivation also positively influences attitudes towards research.

H3b (SN ← CTM): The path from controlled motivation (CTM) to subjective norms (SN) was significant (Unstd. = 0.413, Std. = 0.398, *p* < .001), suggesting a strong positive impact of controlled motivation on subjective norms.

H3c (PBC ← CTM): The path from controlled motivation (CTM) to perceived behavioral control (PBC) was significant (Unstd. = 0.487, Std. = 0.432, *p* < .001), showing that controlled motivation greatly enhances perceived behavioral control.

These results collectively demonstrate that both autonomous and controlled motivations significantly influence the immediate determinants of research intention (attitudes, subjective norms, and perceived behavioral control), which in turn significantly predict the intention to engage in research. Therefore, every path in the hypothesized model was found to be significant and acceptable.

## 5. Discussion

Unlike many studies that center solely on teachers’ teaching practices, this research pivots towards EFL teachers’ research behaviors. By integrating dual theoretical frameworks, we crafted a model that delves into the psychological processes and variables influencing these teachers’ research intentions. This model seamlessly bridges aspects of motivation with the intention for research activities, offering a comprehensive understanding of the determinants guiding college EFL teachers’ research pursuits.

Existing research, as exemplified by studies from Alexander, Wyatt-Smith [60] and Liou, Canrinus [61], underscores the pivotal role of motivation in guiding general teachers’ behaviors. Yet, when narrowing down to the specific context of college EFL teachers, there is a noticeable gap. While a rich body of literature addresses teachers’ motivation, only a few have honed in on the unique motivations driving college EFL teachers, whether autonomous or controlled [e.g., see, 19, 62]. This study bridges this gap by explicitly connecting English teachers’ motivation with their research intention, highlighting an essential yet often overlooked factor in EFL research behavior. In doing so, this study not only builds on existing literature but also outlines significant implications for the realm of teacher education and professional development.

The theoretical model presented in this study harmonizes with prior research findings, bringing together influential factors for English teachers’ research behaviors. Previous research has acknowledged the impact of academic competence, emotions and attitudes, research surroundings, research management, and resources [1, 6]. Within our model, these elements find their alignment: academic competence and resources are tied to English teachers’ perceived behavior control; research surroundings and management correspond to their subjective norms; and emotions and attitudes resonate with teachers’ attitudes towards research.

Expanding beyond alignment, this study offers a significant advancement by integrating SDT and TPB. This novel framework elucidates college EFL teachers’ intention to conduct research in a more comprehensive manner. The findings spotlight the potent roles of both autonomous motivation and controlled the motivation as they substantially influence the proximal predictors of research intention—the three TPB variables: attitudes, subjective norms, and perceived behavior control. This integrated perspective captures the intricate factors related to EFL teachers’ research intention while also illuminating the connections among them, advancing beyond the scope of previous studies that tended to segment these factors [e.g., 3, 4, 6].

From the path coefficients from autonomous and controlled motivation to the three TPB variables, it can be found that the two distal variables have different influences on the three proximal predictors of research intention. Specifically, controlled motivation has more influence than autonomous motivation. This result diverges from numerous previous studies on the interplay between autonomous and controlled motivations. Prior research frequently indicates that autonomous motivation exerts a stronger influence on behavioral intentions, such as research literacy [63] and academic intentions [64, 65]. This might indicate that most college English teachers tend to view research work more as a task to engage in order to receive rewards or avoid punishment than genuinely appreciating the value of research work. This finding echoed with Li and Xu [5]’s research result that extrinsic rather than intrinsic motivation had significant influences on EFL teachers’ research engagement. Therefore, while fostering intrinsic motivation remains important, institutions should prioritize controlled motivation strategies, as our study indicates it has a greater influence on research intentions. Implementing a carrot-and-stick policy can be particularly effective. Tangible rewards such as performance-based incentives, research grants, and public recognition for research achievements can reinforce extrinsic motivation. Additionally, establishing clear expectations and consequences for research productivity can further drive engagement.

This study stands as a pivotal theoretical advancement in teacher education research. By melding SDT and TPB frameworks, it pioneers a nuanced understanding of college EFL teachers’ research motivations and intentions. In line with SDT, our findings reiterate that both autonomous and controlled motivations play instrumental roles in shaping behaviors, echoing the foundational assertions posited by Ryan and Deci [66]. What sets this study apart, however, is the deep dive into how these motivations interface with TPB constructs. Our findings not only bridge a gap in the TPB literature but also illuminate the intricate ways through which motivation molds the proximal predictors of research intention, namely attitudes, subjective norms, and perceived behavioral control. This confluence of SDT and TPB offers a comprehensive lens, highlighting the multifaceted interplay between motivation and behavioral intentions. Consequently, the theoretical scaffolding presented in this research serves as an imperative guide for future studies and intervention designs aiming to foster enhanced research intentions among educators.

## 6. Conclusion and implications

In conclusion, the paramount objective of this study was to craft a robust theoretical framework, seamlessly integrating the tenets of SDT and TPB, aimed at elucidating the intricacies of college EFL teachers’ research intentions. Specifically, it illuminated how both autonomous and controlled motivations substantially influence the proximal predictors (attitudes, subjective norms, and perceived behavior control) of research intention. Furthermore, these motivations exhibited indirect effects on college EFL teachers’ research intentions, mediated by the TPB variables. The synthesized framework, marrying SDT and TPB, stands as a beacon for designing targeted interventions to invigorate teachers’ intrinsic drive and intentions for research, thereby bolstering research behavior among college EFL teachers. The findings have profound implications for teacher education programs and professional development initiatives.

Firstly, the significance of nurturing teachers’ controlled motivation for research is underscored in this study. In light of this, prescribing specific research assignments to college EFL teachers emerges as a strategic lever to amplify their research inclinations. These assignments can be designed to align with the teachers’ existing workload and research interests. For example, colleges can set clear timelines and milestones for research projects, provide regular inspection, feedback and support from research mentors and communities. Higer education institutions could also implement carrot and stick research policies among EFL teachers: providing financial incentives such as research grants, bonuses, or additional funding for research-related activities based on research output and performance. Simultaneously, clear consequences for failing to meet research expectations should be communicated. For instance, teachers who do not meet research requirements could face penalties such as reduced opportunities for promotion, salary reductions, or fines. Importantly, these strategies should be adapted to suit the diverse contexts of different colleges. Public ones might focus on leveraging their typically larger resources to offer more substantial incentives and support structures, whereas private institutes could emphasize more personalized support and flexible research assignments to cater to their unique environments.

Concurrently, the role of autonomous motivation shouldn’t be overlooked, as it has a tangible impact on shaping research intentions as well. Teacher education initiatives should accentuate the intrinsic benefits of research for both personal and professional evolution, while concurrently offering avenues for teachers to delve into research endeavors resonating with their unique interests and aspirations. Through the cultivation of autonomous motivation, teachers are poised to harbor a more favorable disposition towards research, gain a heightened sense of agency over their research undertakings, and draw encouragement from peers and institutional frameworks. Thus, a balanced approach, one that champions both facets of motivation, stands to be the most potent catalyst in augmenting research engagement among college EFL educators.

Additionally, in light of the pronounced influence of subjective norms and perceived behavioral control on research intention, it becomes imperative for professional development endeavors to pivot towards the creation of conducive research atmospheres and to furnish resources that streamline research practices. Educational establishments might consider instituting dedicated research collectives or communities of practice to galvanize collaborative efforts and extend robust backing for research initiatives. Furthermore, to sustain motivation and research engagement over the long term, institutions should offer continuous professional development opportunities and maintain ongoing support systems like mentorship programs. Establishing mechanisms and environments for recognizing and rewarding sustained research efforts, such as long-term grant funding and research excellence awards, can help keep teachers engaged and motivated in their research endeavors. Such provisions can significantly bolster teachers’ research intentions and assurance.

## Supporting information

S1 Dataset Original data. (XLS)
